# Ka Band Holographic Imaging System Based on Linear Frequency Modulation Radar

**DOI:** 10.3390/s20226527

**Published:** 2020-11-15

**Authors:** Yang Meng, Chuan Lin, Jiefeng Zang, Anyong Qing, Natalia K. Nikolova

**Affiliations:** 1 School of Physics, University of Electronic Science and Technology of China, Chengdu 611731, China; mengyang@std.uestc.edu.cn; 2 School of Electrical Engineering, Southwest Jiaotong University, Chengdu 611756, China; linc@swjtu.edu.cn (C.L.); zjf@swjtu.edu.cn (J.Z.); 3 Department of Electrical and Computer Engineering, McMaster University, Hamilton, ON L8S 4K1, Canada; nikolova@ieee.org

**Keywords:** holographic imaging, linear frequency modulation, millimeter wave, security check

## Abstract

Millimeter wave (MMW) technology is expanding rapidly into security screening for dangerous items concealed under clothing. It uses safe non-ionizing radiation and penetrates clothing well. We present a new planar system at Ka band for the three-dimensional simultaneous imaging of both sides of an inspected person where the images are produced in real time by a recently proposed generalized holographic reconstruction algorithm. Low-cost linear frequency modulation (LFM) radar technology is used along with a simple but efficient method for system calibration. Experimental characterization of the spatial resolution and the sensitivity of the system prototype has been carried out. It is established that the achieved spatial resolution is 6 mm or better if the item is not obscured by clothing and it may deteriorate to 7 mm depending on the clothing hiding the item. The spatial sensitivity is confirmed to be at least 2 mm.

## 1. Introduction

In the last two decades, the threat of terrorism and armed violence has become a major concern around the world [1,2,3]. Various engineering solutions are being developed to ensure the safety of people and their property. At present, the focus is on protecting public places and especially transportation hubs, such as airports and railway and bus stations, by preventing people from bringing in weapons and explosives concealed under their clothing or in their luggage.

There are various methods for security inspection [4,5,6,7], ranging from security presence (e.g., police personnel and dogs) through handheld and walk-through metal detectors [8] to sophisticated whole-body scanners (millimeter wave and X-ray) and emerging radars for concealed weapon detection (CWD) on moving people [9]. However, the reliability and efficiency of these methods are insufficient to ensure the public’s safety in an ever-changing spectrum of threats. Metal detectors are incapable of detecting plastics, liquids, and ceramics, which materials are often used to fabricate explosives, handheld weapons, and contraband items. However, non-metallic objects are detectable by technologies that use higher frequencies such as millimeter waves, infrared, and X-rays. Unfortunately, the infrared technology [8,10] suffers from poor penetration through thick clothing whereas X-ray imagers use ionizing radiation, which is not suitable for deployment in public areas due to health hazard.

Millimeter-wave imaging technology has proven to be a safe and effective means for the detection of concealed items on the human body. It has emerged as the leading technology for the future of the security screening of people [11,12,13,14,15,16,17,18,19,20]. Two types of millimeter wave imaging systems (MMWIS) have been developed: active (AMMWIS) and passive (PMMWIS). The AMMWIS appear to have better detection capability in terms of higher sensitivity and robustness to environmental noise and interference. One of the most efficient real-time reconstruction methods employed by the AMMWIS in whole-body imaging is microwave holography [21,22,23]. Heterodyne mixing in the receivers ensures amplitude and phase information in the back-scattered signal from the object of interest, which allows for three-dimensional (3-D) imaging within seconds.

Here, we propose a radar system based on linear frequency modulation (LFM) radars, which enables faster measurements than that with stepped frequency radars. The generalized holographic imaging with LFM (GHI-LFM) method has also been developed to process the LFM signals [24]. The Ka-band in-house prototype has been first presented in [25]. Here, we focus on the hardware design of the imaging system developed for verifying the GHI-LFM inversion theory, and the design of its modules, on the improved calibration method, and on the various experimental characterizations, including characterization of its spatial resolution and sensitivity, the effects of different clothing materials. Finally, tests with volunteers carrying various concealed objects indicate excellent imaging capability in real-life deployment for whole-body screening.

The GHI-LFM reconstruction algorithm is briefly introduced in Section 2. Details of the in-house 3-D imaging prototype are given in Section 3 whereas an improved system calibration method is described in Section 4. Results of the experiments with human volunteers and various concealed items are given in Section 5. Conclusions are drawn in Section 6.

## 2. A Brief Description of the GHI-LFM Algorithm

**Figure 1:**
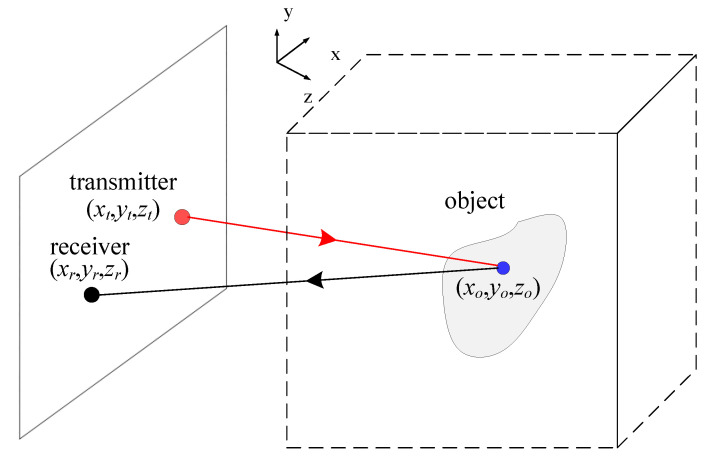
Setup of the planar holographic imaging system.

The configuration of the general holographic imaging system is shown in Figure 1. The object of interest is represented by its scattering coefficient σ(**r***_o_*), where **r***_o_* = (*x_o_*, *y_o_*, *z_o_*) is a point within the object. The transmitter at **r***_t_ =* (*x_t_*, *y_t_*, *z_t_*) emits the LFM millimeter wave toward the object while the receiver at **r***_r_ =* (*x_r_*, *y_r_*, *z_r_*) receives the echo from the object. For simplicity, a monostatic scenario is assumed, i.e., **r***_r_* = **r***_t_*.

The de-chirped and compensated echo signal is modelled [24] by

(1) $$s\left(r_{r},\hat{t}\right)=∭\frac{\sigma (r_{o})}{R}P\left(\frac{\hat{t}-2R/c}{T_{p}}\right)\exp\left[-i2\pi \frac{2R}{c}\left(f_{c}+\gamma \hat{t}\right)\right]dr_{o}$$

where $\hat{t}$ is the fast time in each LFM period, *T_P_* is the pulse width, *f_c_* is the center frequency, *γ* is the chirp rate, *c* is the speed of light, *R* is the distance from the antennas to the object, and

(2) $$P\left(u\right)=\left\{ \begin{array}{l} 1,\;|u|\leq 1/2\\ 0,\;\mathrm{otherwise}. \end{array}\right.$$

Note that a time factor *exp(-iωt)* is assumed and suppressed, where *i* is imaginary unit, *ω* is the angular frequency, and *t* is time. (1) can be expressed as

(3) $$s\left(r_{r},k\right)=∭\frac{\sigma (r_{o})}{R}\exp\left(-i2kR\right)dr_{o}$$

with $k=2\pi \left(f_{c}+\gamma \hat{t}\right)/c,\;-T_{p}/2+\frac{2R}{c}\leq \hat{t}\leq T_{p}/2+\frac{2R}{c}$, and

(4) $${\left(2k\right)}^{2}=k_{x}^{2}+k_{y}^{2}+k_{z}^{2}\;.$$

The scattering coefficient *σ*(**r**_o_) is retrieved as

(5) $$\sigma \left(r_{o}\right)=FT_{\left(k_{x},k_{y},k_{z}\right)}^{-1}\left\{FT_{\left(x_{r},y_{r}\right)}\left[s\left(r_{r},k\right)\right]\frac{ik_{z}e^{-ik_{z}z_{r}}}{2\pi }\right\}.$$

where $FT_{\left(x_{r},y_{r}\right)}$ is the two-dimensional (2-D) Fourier transform (FT) with respect to spatial variables (*x_r_*, *y_r_*) and $FT_{\left(k_{x},k_{y},k_{z}\right)}^{-1}$ is the 3-D inverse FT with respect to spectral-frequency variables (*k_x_*, *k_y_*, *k_z_*).

## 3. In-house Prototype System

In this section, an in-house prototype of a 3-D millimeter wave imaging system based on GHI-LFM is introduced. The key parameters of the system are listed in [24].

### 3.1. System Framework

**Figure 2:**
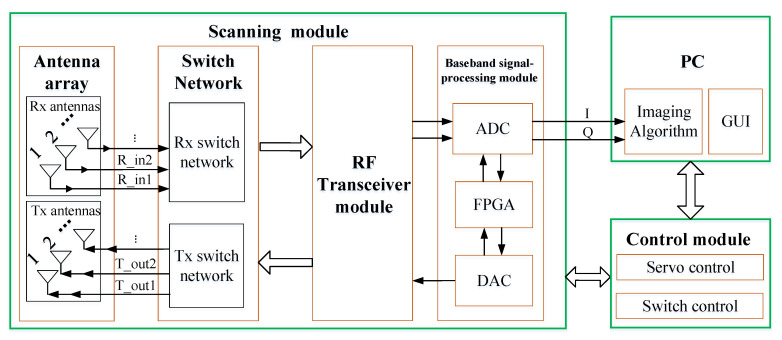
Block diagram of the imaging system prototype.

**Figure 3:**
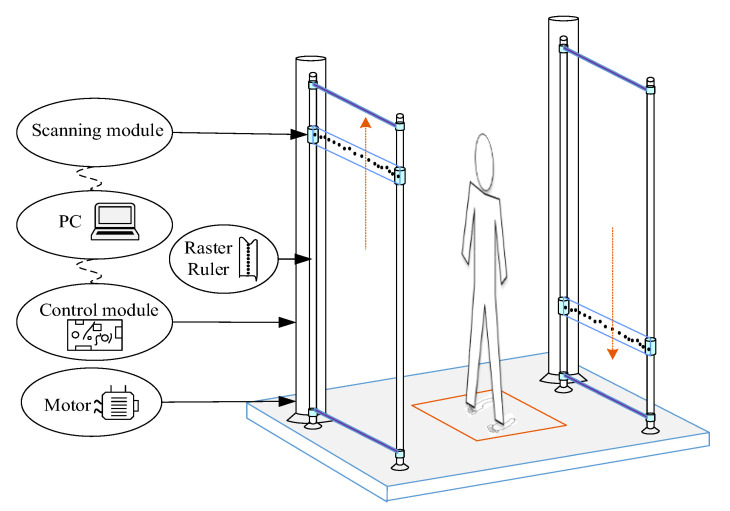
Framework of the imaging system prototype.

As shown in Figure 2 and Figure 3, the system is composed of a personal computer (PC), a control module and a scanning module. The imaging algorithm and a graphic user interface (GUI) are implemented in the PC. The GUI sets the system control parameters, initializes the system, and triggers scanning. The object of interest is reconstructed by the GHI-LFM and the image is displayed on the PC monitor. The control module includes servo and switch controls for the mechanical (vertical) and electrical (horizontal) scanning of the system. The scanning module consists of the baseband module, an RF transceiver module, two RF switch networks and two antenna arrays. The baseband signal-processing module consists of an analog to digital converter (ADC), a digital to analog converter (DAC) and a signal-processing unit based on a field-programmable gate array (FPGA). The RF transceiver module contains the millimeter-wave source, providing the signal to the transmitting (Tx) antennas. It also receives the echo signal from the receiving (Rx) antennas. Figure 2 shows the block diagram of the system, whereas Figure 3 illustrates its setup and framework.

### 3.2. Transceiver Antenna Array

**Figure 4:**
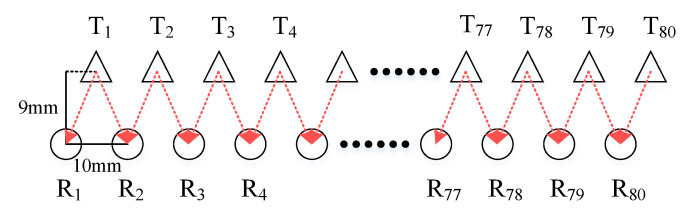
The configuration of the scanning antenna array.

The imaging system uses 2-D planar scanning to reconstruct a 3-D image of the object of interest. The transceiver antenna array is horizontal, and it is controlled by an RF switch network to accomplish electronic scanning in the horizontal direction. For sequential uniform scanning, the Tx and Rx antennas are staggered within a 10-mm separation distance in the horizontal direction and a 9-mm separation in the vertical direction as shown in Figure 4. There are 80 Tx antennas in the upper row and 80 Rx antennas in the lower row, with 5 mm interleaving in the horizontal direction. The RF switches control the feeds of transmitting and receiving antennas. For each antenna in the array, the antenna gain is about 9 dBi and the measured –3 dB azimuthal beamwidth is around 58° to 64° whereas the elevation one is around 55° to 63°, depending on the frequency from 27.0 GHz to 32.8 GHz. Only one pair of Tx and Rx antennas is activated at any given time, starting with the pair T_1_-R_1_ and ending with the T_80_-R_80_ pair. Note that each Tx antenna is paired with two neighboring Rx antennas, shown by the red arrows in Figure 4. Thus, the total number of collected signals is 159.

The vertical scan is accomplished by a servo motor with a constant sampling interval of 5 mm. The scanning array moves vertically to the next position only after the electronic scanning in the horizontal direction is completed. The total vertical travel distance is 1.9 m, i.e., there are 380 rows in total. Thus, the number of spatial sampling positions is 380 × 159. At the same time, the number of temporal sampling points per LFM sweep at every position is 200. So, the format of sampled echo data is 380 × 159 × 200.

### 3.3. RF Switch Network

**Figure 5:**
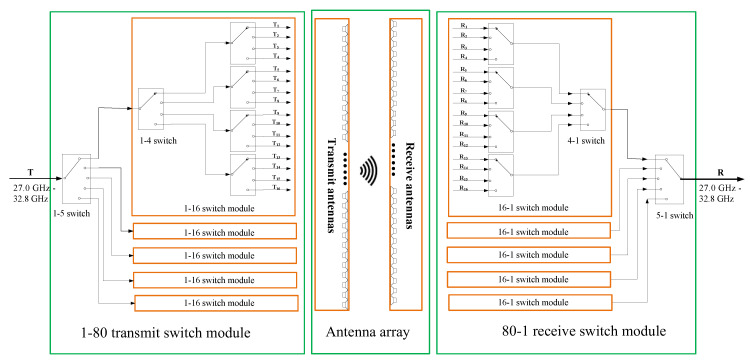
The RF switch network.

As shown in Figure 5, the RF switch network includes a 1-to-80 Tx switch module and an 80-to-1 Rx switch module. The Tx switch module is a corporate arrangement of 1-5-4-4. The Rx switch module employs the same arrangement in reversed order. The RF switch components are: (i) SP5T (single pole, 5 throw) switch MA4AGSW5 [26], (ii) SP4T switch MA4AGSW4 [27].

### 3.4. RF Transceiver Module

**Figure 6:**
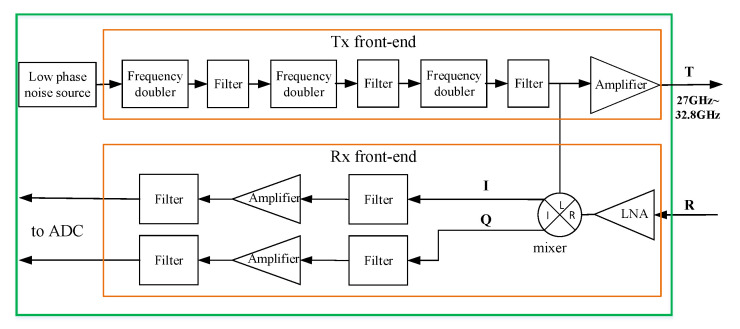
Block diagram of the RF transceiver module.

In the RF transceiver module (see Figure 6), the Tx branch carries out 8-fold frequency up-conversion of the signal generated by the FPGA up to 27.0–32.8 GHz. It also carries out filtering and amplification. In each Tx branch, the minimum power emitted from Tx antenna is over 0 dBm. In actual imaging tests, the transmitting power between 15 dBm to 20 dBm is fine enough.

The echo signal received by an Rx antenna is amplified by a low noise amplifier (LNA) and is then mixed with the local-oscillator signal branched out from the Tx front-end. Two intermediate frequency (IF) signals, I and Q, are obtained. They are filtered and amplified separately before being submitted to the baseband signal-processing module. The noise figure is designed to maintain below 16 dB and the corresponding receiver sensitivity can be better than −80 dBm at room temperature.

### 3.5. Baseband Signal-Processing Module

**Figure 7:**
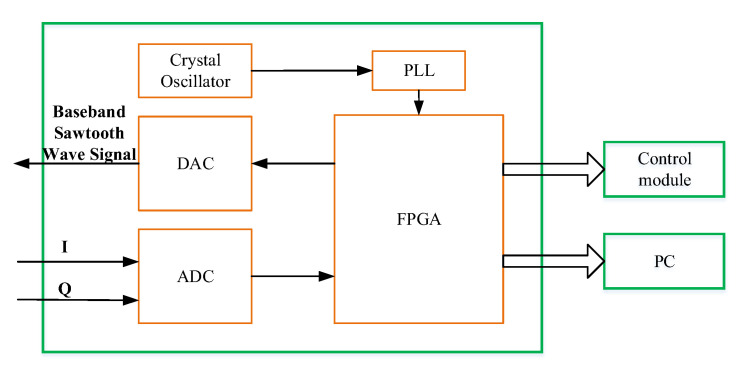
Block diagram of the Baseband signal-processing module.

The baseband signal-processing module performs the following tasks: (i) generates the Tx saw-tooth baseband signal, (ii) receives and pre-processes the IF signals, (iii) forwards the processed signals to the PC and the control module, (iv) issues control commands, and (v) synchronizes the clocks of the imaging system. Key components of this module include crystal oscillator, phase-locked loop (PLL), DAC, ADC, and FPGA. The baseband saw-tooth signal is realized by the FPGA and the DAC whereas the signal acquisition is performed by the FGPA and the ADC. The system control and the signal preprocessing are done by the FPGA. Figure 7 shows the block diagram of the baseband module.

### 3.6. In-House Developed Prototype

**Figure 8:**
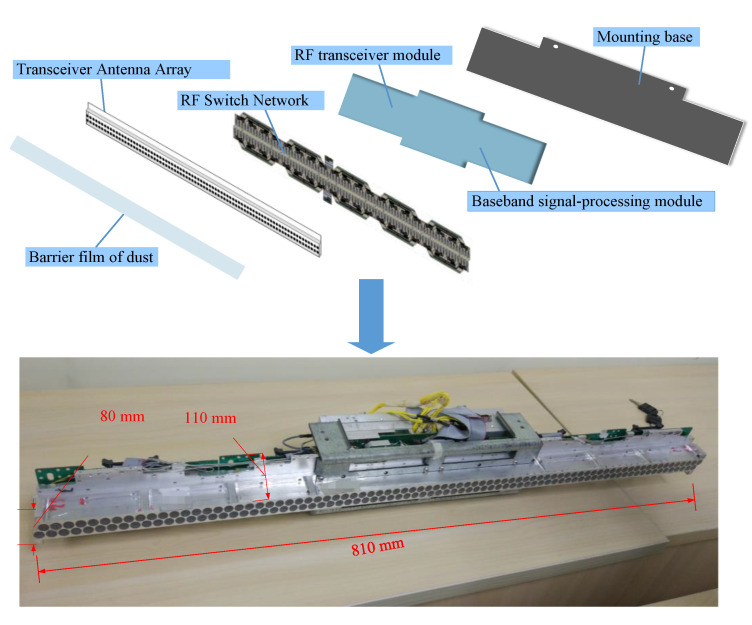
Photo of the dismantled and integrated LFM radar module.

**Figure 9:**
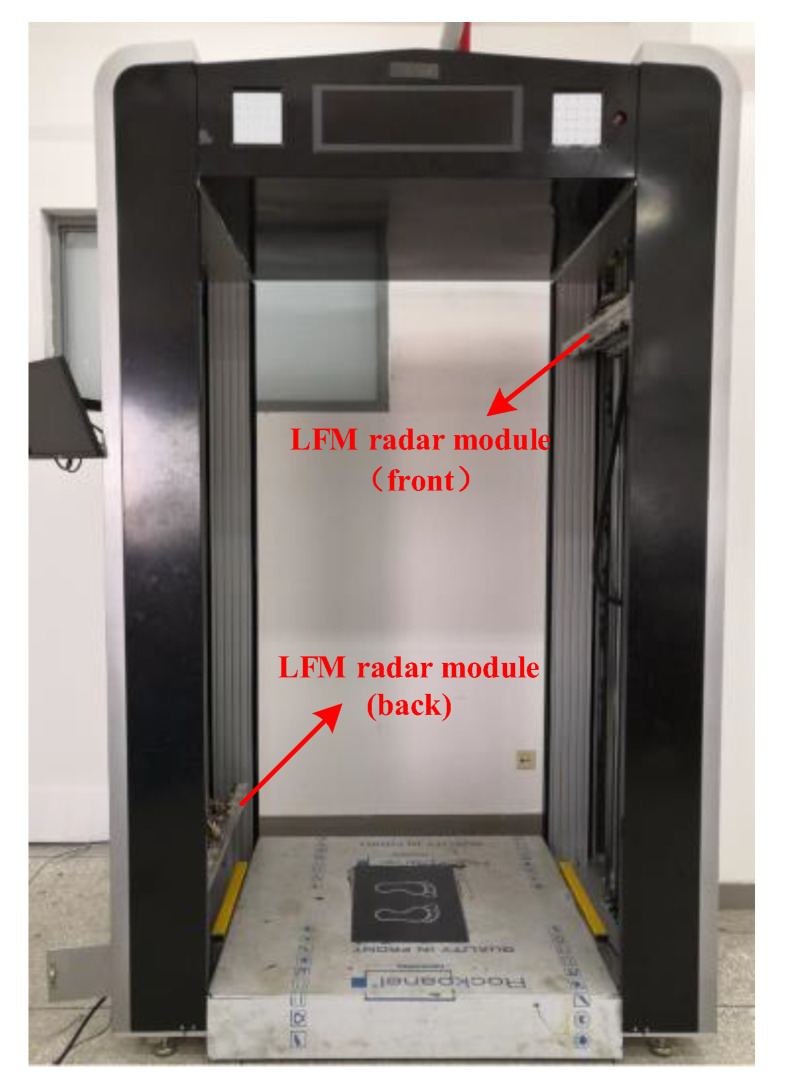
Photo of the in-house developed prototype.

All the modules presented above, including transceiver antenna array, RF switch network, RF transceiver module, baseband signal-processing module, are integrated together in a complete LFM radar module in accordance with the carefully designed layout, as shown in the photo in Figure 8. It is integrated in a small volume, with the size of 810 mm × 110 mm × 80 mm, convenient for assembly, disassembly, and transport. Figure 9 shows a photo of the imaging prototype system. Two LFM radar modules are assembled on both sides of the prototype. The scanning area is 0.8 m × 1.9 m. When a person stands within the test area, the scanning arrays at the front and the back work synchronously but the scanning directions are opposite, that is, when the front array sweeps from top to bottom, the back array sweeps from bottom to top, or vice versa. Similarly, in the horizontal scanning, the directions are also opposite, that is, when the front array sweeps from left to right, the back array sweeps from right to left, or vice versa. This scanning scheme minimizes the interference between the front and back arrays while they work simultaneously. Both sides of the person under test are scanned within 2 s. The GHI-LFM algorithm is programmed for parallel execution using CUDA C language on an NVIDIA GTX 1080Ti GPU platform. A 3-D image is obtained within 1 s by the GHI-LFM. This imaging time is comparable or better in comparison with previously reported systems [21,28,29].

## 4. System Calibration

The system adopts vertical mechanical scanning, horizontal electronic scanning and broadband LFM scanning, each being susceptible to various types of measurement uncertainty and noise. The down-converted echo signal is decomposed into *I* and *Q* components by the harmonic mixer [30]. The inconsistency among the 159 RF channels and the unbalance between the *I* and *Q* branches in each channel must be calibrated before the imaging processing.

**Figure 10:**
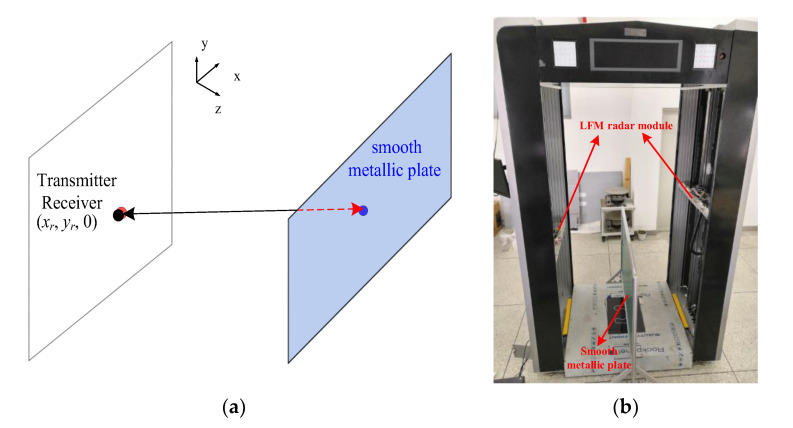
(**a**) Schematic diagram of a system calibration measurement, (**b**) photo of the system calibration scene.

The authors [24] presented a simple and effective calibration scheme to deal with the measurement uncertainty and noise of the imaging system by measuring the actual echo signals for every channel and calibrating each other individually. In this paper, we adopt a simpler but more effective calibration scheme, depicted in Figure 10a. It employs a flat metallic plate serving as an ideal reflector, which is fixed at the plane z*_o_* = 0.4 m with the plate center aligned with the center of the antenna array. For each antenna in the array, according to the measured −3 dB beamwidth, the largest beam spot on the plate does not exceed 0.5 m. The fabricated metallic plate is 1 m in width and 0.5 m in height, as shown in Figure 10b. It is assumed to have a reference scattering coefficient $\sigma (x_{o},y_{o})=1,$ ignoring the phase reversal upon reflection. The theoretical echo data for calibration is calculated using (1) only for the channel corresponding to the middle receiving antenna as

(6) $$s_{t}\left(\hat{t}\right)=\iint_{S_{M}}\frac{1}{R}P\left(\frac{\hat{t}-\frac{2R}{c}}{T_{p}}\right)\exp\left[-i\frac{4\pi R}{c}\left(f_{c}+\gamma \hat{t}\right)\right]dx_{o}y_{o}$$

where *S_M_* is the surface area of the metallic plate within the −3 dB antenna beamwidth.

Assume that the truly measured echo data by each channel is $s_{m}(r_{r},\hat{t})$. For all measured channels, the theoretical echo data would be obtained by (6). The amplitude calibration coefficient for this channel is calculated as the ratio of the peak signal strengths in frequency spectrum between this channel and the middle channel: $A_{tp}=\max\left\{\left|FT\left[s_{t}(\hat{t})\right]\right|\right\}$ and $A_{tm}\left(r_{r}\right)=\max\left\{\left|FT\left[s_{m}(r_{r},\hat{t})\right]\right|\right\}$, i.e.,$m_{\mathrm{cal}}\left(r_{r}\right)=A_{tp}/A_{tm}\left(r_{r}\right).$ The time-dependent phases $\varphi_{t}(\hat{t})$ and $\varphi_{m}(r_{r},\hat{t})$ of $s_{t}(\hat{t})$ and $s_{m}(r_{r},\hat{t})$, respectively, are subtracted to obtain the phase correction $\varphi_{\mathrm{cal}}(r_{r},\hat{t})=\varphi_{t}(\hat{t})-\varphi_{m}(r_{r},\hat{t})$. Finally, the correction is regarded as a standard compensation to be applied to corresponding channels during actual measurements using

(7) $$s_{\mathrm{cal}}(r_{r},\hat{t})=m_{\mathrm{cal}}\left(r_{r}\right)s(r_{r},\hat{t})\cdot \exp\left[i\varphi_{\mathrm{cal}}(r_{r},\hat{t})\right].$$

The above IF signals are submitted to the GHI-LFM algorithm for image reconstruction. Compared with the calibration procedure recently proposed in [24] or other conventional calibration methods [28], where echo signals of every channel are simulated and measured to calibrate each other individually, here, only one channel theoretical echo data(corresponding to the central antenna pair in the array) is used. The so obtained magnitude and phase corrections are then used on corresponding channels. This calibration method is simpler, faster, and suitable for system calibration after deployment. It is confirmed valid by the later measured data.

## 5. Test Result and Discussion

The prototype system has been tested and validated through numerous measurements. Its spatial resolution and sensitivity have been quantitatively characterized, followed by the qualitative imaging of volunteers and various objects. Although the system poses no radiation hazard to the human body, informed consent was obtained from all volunteers.

### 5.1. Spatial Resolution

The image spatial resolution is an essential performance metric of the imaging algorithm and the whole system. It is defined as the minimal space between two benchmark objects at or below which the two objects are not distinguishable in the reconstructed image. Here, we use groups of metallic strips as benchmark objects to establish experimentally the system spatial resolution limits.

**Figure 11:**
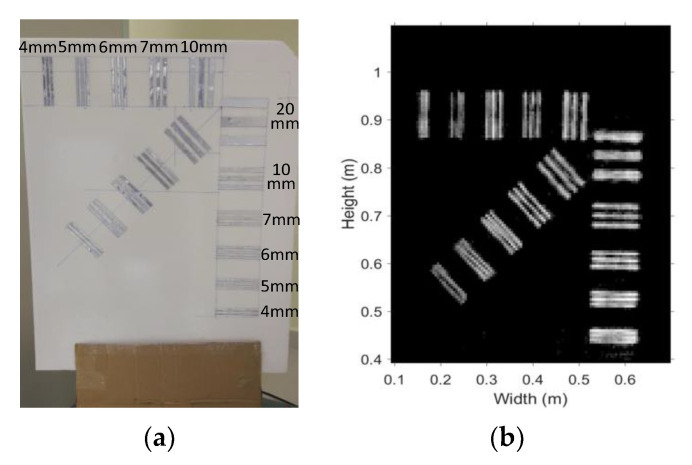
Spatial Resolution Board (**a**) Photo, (**b**) MMW Image.

The benchmark-target board for testing the system spatial resolution is shown in Figure 11a, which is similar to the board used in [24], but the orientation of the target board is opposite. The MMW image of the board is given in Figure 11b. The strips of 5 mm and wider in the oblique group can be clearly recognized. However, only strips of 6 mm and wider in both the horizontal and vertical groups are clearly recognized.

**Figure 12:**
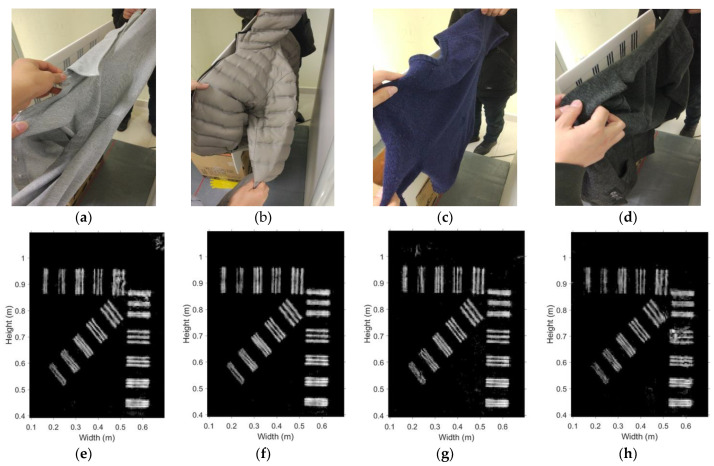
Effect of fabrics blocking the target: (**a**) shirt, (**b**) down jacket, (**c**) sweater, (**d**) woolen coat. The respective images are shown in (**e**–**h**).

Qualitative test of the blocking effect of fabrics has also been conducted. A shirt, a down jacket, a sweater, and a woolen coat are used to cover the benchmark-target board sequentially, as shown in Figure 12a–d. The corresponding images are shown in Figure 12e–h. Slight deterioration is observed when the board is covered by the woolen coat. Otherwise, the blocking effect is negligible.

The results for the system spatial resolution limits in the various test scenarios are summarized in Table 1. It is evident that all clothing materials are transparent for the MMWs from 27.0 GHz to 32.8 GHz. The worst spatial resolution is 7 mm in the case of the woolen coat covering the target. The best resolution of 5 mm is in the diagonal direction. These limits agree with the theoretical limit of half-wavelength. The working frequency band of the LFM system is from 27.0 GHz to 32.8 GHz, which corresponds to a spatial resolution limit of about 4.6 mm (one-half of the shortest available wavelength).

### 5.2. Spatial Sensitivity

**Figure 13:**
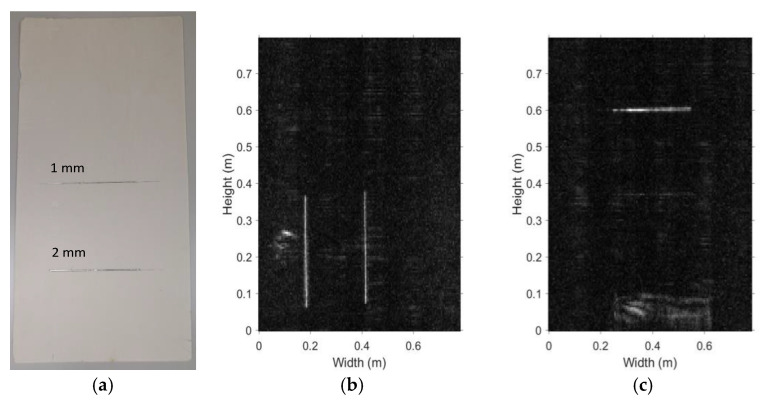
Sensitivity test board: (**a**) photo of the board, (**b**) MMW image of the board placed vertically, (**c**) MMW image of board placed horizontally.

The spatial sensitivity is another important metric of system performance. In our experiments, it is defined as the width of the thinnest single metal strip at or below which the system is not able to image the metal strip. As shown in Figure 13a, the sensitivity test board consists of two tinfoil strips glued on a Styrofoam board, placed parallel to the scanning plane at the distance of 0.4 m. The width of each strip is 1 mm and 2 mm, respectively. Both strips are 300 mm long.

Considering the linear polarization of the antennas, the sensitivity test board is placed so that the tinfoil strips are oriented horizontally and then vertically relative to the antenna array in two separate experiments. The respective images are shown in Figure 13b,c. The results indicate that both strips are clearly visible in the vertical placement scenario whereas in the horizontal scenario only the 2 mm strip can be clearly detected. We conclude that the system sensitivity is limited to an object size of about 2 mm. As is expected from a system with low measurement uncertainty and noise, the system spatial sensitivity is limited to objects smaller than the spatial resolution limit.

### 5.3. Tests with Emulated Contraband Samples

**Figure 14:**
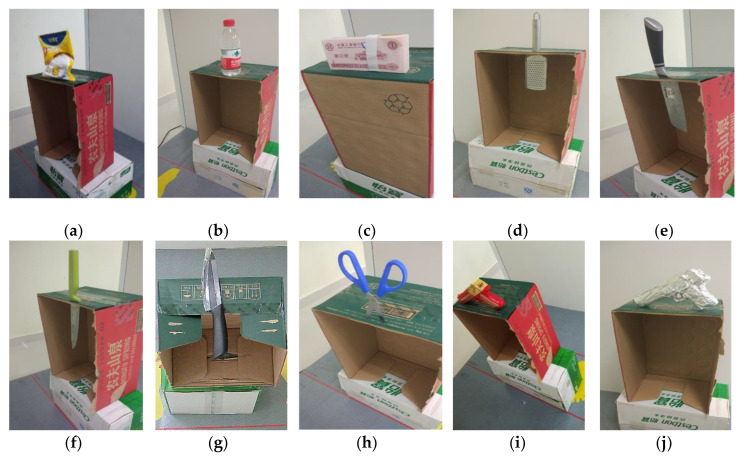
Emulated contraband samples used to test the detection capability of the imaging prototype: (**a**) bagged starch, (**b**) bottled water, (**c**) banknotes, (**d**) metallic grater, (**e**) kitchen knife, (**f**) metallic fruit knife, (**g**) ceramic knife, (**h**) scissors, (**i**) plastic handgun, (**j**) metallic handgun.

Figure 14a–j shows the various emulated contraband samples used to test the system. These include bagged starch (18 cm × 16 cm × 2 cm), bottled water (6 cm in diameter and 18 cm in height), banknotes (100 pcs, 14 cm × 6.4 cm × 1.45 cm), metal grater, kitchen knife, metallic fruit knife, ceramic knife (about 1 mm thick), scissors, plastic handgun, and metallic handgun (the plastic handgun wrapped in tinfoil). The objects are placed on cartons one by one and their images are generated to test the detection capability of the prototype.

**Figure 15:**
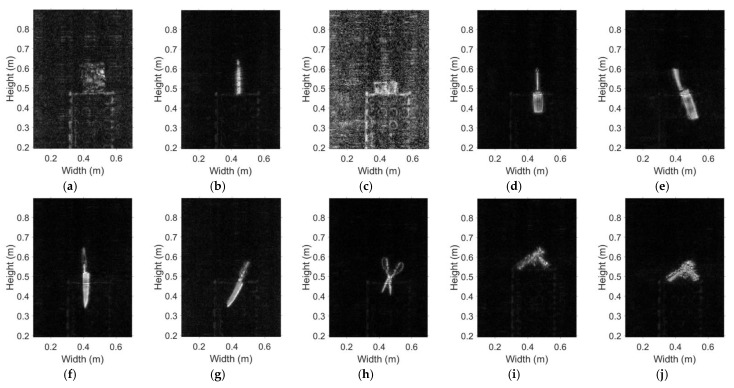
MMW images of the emulated contraband items, the photos of which are shown in Figure 14: (**a**) bagged starch, (**b**) bottled water, (**c**) banknotes, (**d**) metallic grater, (**e**) kitchen knife, (**f**) metallic fruit knife, (**g**) ceramic knife, (**h**) scissors, (**i**) plastic handgun, (**j**) metallic handgun.

The respective MMW images of the samples are shown in Figure 15a–j. We can see that the metallic items such as the grater, the knife, the scissors, and the handgun are reconstructed very clearly. The images of the ceramic knife, plastic handgun, and the bottled water are also very clear. The bagged starch and the banknotes appear faintly in the images. Note that the noise in all experiments is at the same level. Since the reflectivity of the banknotes and bagged starch are low and similar to those of the cardboard box and the background, the “noise” in the images becomes apparent, which indicates that they may be more difficult to detect on the human body.

### 5.4. Screening of Volunteers with Hidden Contraband Samples

**Figure 16:**
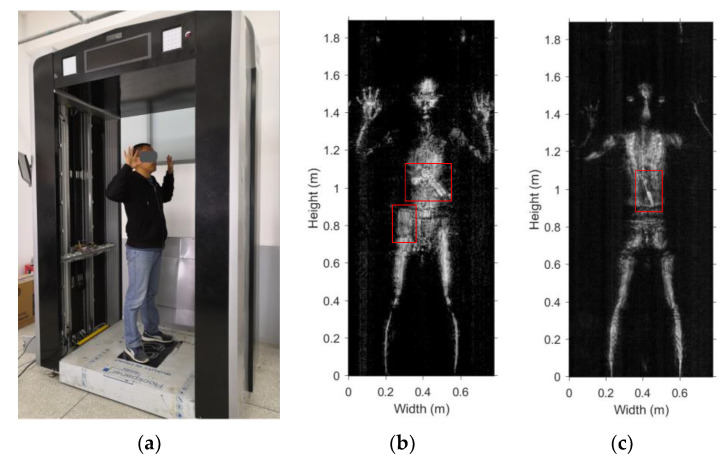
Volunteer with hidden contraband items: (**a**) screening scenario photo, (**b**) MMW image of the front of the volunteer, (**c**) MMW image of the back.

Members of the R&D team have volunteered in tests of the imaging prototype in realistic scenarios of people carrying various objects under their clothing. The volunteers wore a black down jacket and hid the contraband samples underneath. Also, a cell phone was present in the side pocket. The screening scenario is shown in Figure 16a. Both sides of the volunteer’s body are imaged simultaneously. The front and back images are shown in Figure 16b,c. The front image clearly shows a gun-shaped item at the chest and a cellphone-like item at the upper thigh. The back image detects the fruit knife.

**Figure 17:**
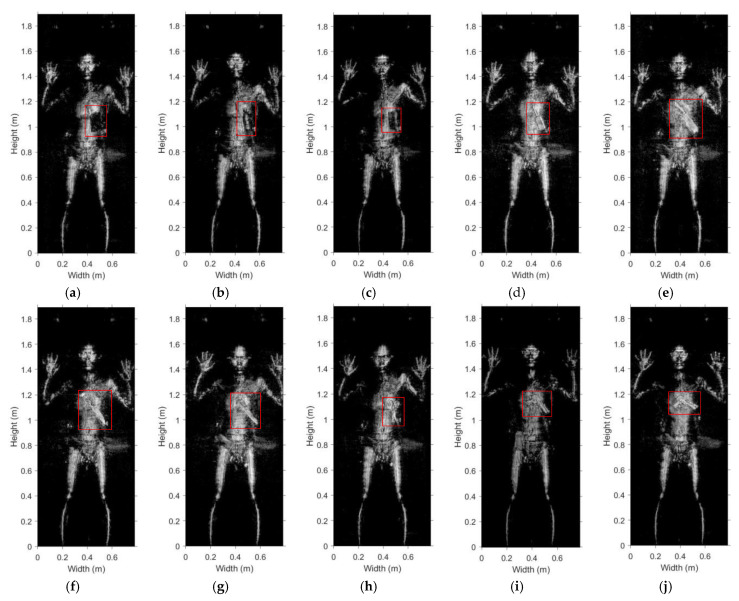
MMW images of a volunteer hiding under a down jacket: (**a**) bagged starch, (**b**) bottled water, (**c**) banknotes, (**d**) metallic grater, (**e**) kitchen knife, (**f**) metallic fruit knife, (**g**) ceramic knife, (**h**) scissors, (**i**) plastic handgun, (**j**) metallic handgun.

In further experiments, the contraband items described in the previous subsection (see also Figure 14) are hidden one by one in a thick down jacket on a volunteer’s body and are imaged. The corresponding MMW images are shown in Figure 17a–j, among which bottled water and ceramic knife are first presented in [24]. The imaging results show that all the contraband items can be identified, no matter metallic or non-metallic. The bagged starch and banknotes appear darker than the human body, suggesting significant absorption. The middle part of the bottled water is brighter than its edges, which is due to the differences in the direction of scattering. The reflectivity of the ceramic knife, while smaller than that of a metallic knife, is sufficient to exceed the brightness of the human body. Note that in images in Figure 17a,b,d–h,j, we can find a coin in the person’s pocket (upper portion of thigh on the right). Even the facial expressions of the volunteer can be recognized to some extent.

## 6. Conclusions

We presented a 3-D Ka band holographic imaging system implementing simultaneous 2-D planar scanning on both sides of an inspected person and generating in real time the respective images using the effective GHI-LFM reconstruction algorithm. A staggered transceiver antenna array is developed which is controlled by an RF switch network. A simple, economical, and effective calibration approach is proposed based on a calibration measurement with a metallic plate.

The spatial resolution of the prototype system has been quantitatively studied and found to be 5 mm to 6 mm depending on the orientation of the object. The impact of various clothing materials has also been investigated and has been found to be negligible. A preliminary characterization of the spatial sensitivity of the system has been conducted, indicating that the system is capable of detecting objects as small as 2 mm. This conclusion is confirmed in experiments with volunteers hiding various object under clothing. Not only are the larger objects identified well, but a small coin in the front pocket is also detected.

The prototype system has been tested with various objects and contraband samples, including bagged starch, bottled water, banknotes, metallic grater, kitchen knife, metallic fruit knife, ceramic knife, scissors, plastic gun, and metallic gun. The experimental results show that all metallic objects as well as the ceramic knife, plastic gun and the bottled water can be detected very clearly under any type of clothing. The detection of the bagged starch and the banknotes is the most challenging, yet, tests with volunteers carrying these objects under a thick jacket demonstrate that such detection is possible. Overall, the specifically designed Ka band holographic imaging system based on LFM radar verifies the efficiency of the GHI-LFM inversion theory and proves to be a very promising candidate for the upcoming generation of whole-body screening technologies.

## Figures and Tables

**Table 1:** Results of the system spatial resolution.

Covers	Horizontal	Vertical	Oblique
Uncovered	6 mm	5 mm	5 mm
Shirt	6 mm	6 mm	5 mm
Down jacket	6 mm	5 mm	5 mm
Sweater	6 mm	6 mm	5 mm
Woolen coat	7 mm	6 mm	5 mm
Uncovered	6 mm	5 mm	5 mm
